# Study protocol and baseline characteristics of newly induced dialysis patients: a prospective multi-center cohort study with a biological sample bank, the Ibaraki Dialysis Initiation Cohort (iDIC) study

**DOI:** 10.1186/s12882-022-02729-3

**Published:** 2022-03-15

**Authors:** Takashi Tawara-Iida, Joichi Usui, Itaru Ebihara, Takashi Ishizu, Masaki Kobayashi, Yoshitaka Maeda, Hiroaki Kobayashi, Tokuro Kobayashi, Atsushi Ueda, Makoto Tsuchida, Shinichiro Sakai, Kunihiro Yamagata, and the Study Group of the Ibaraki Dialysis Initiation Cohort Study, Hideko Nakamura, Kenji Takada, Koichi Kozaki, Satoshi Iwabuchi, Tadashi Iitsuka, Kenta Nishiki, Hideaki Takasaki, Takashi Takita, Masami Nakajima, Sumiko Honma, Youichi Akai, Genzou Ishizuka, Koichi Issiki, Takako Saito, Hitoshi Iwamoto, Akira Ohishi, Masakazu Ohtsuka, Atsushi Ono, Hidehiko Kashiwabara, Takuro Kanekawa, Naoaki Kanamori, Fumika Kaneda, Hiroshi Kikuchi, Masashi Kubo, Hiromi Kurosawa, Takeshi Shiraishi, Tatsuo Shiigai, Masayoshi Shima, Tokuo Takahashi, Hideki Matsukawa, Minoru Tokoi, Sadao Tsunematsu, Atsushi Tsuruta, Masao Deguchi, Masahiro Hayakawa, Makoto Hiroi, Nobuki Maeda, Takanobu Hoshino, Tetsu Yamaguchi, Kota Yamada, Atsushi Takeda, Ikuo Takahashi, Takamichi Yuhara, Tadashi Kondo, Syoji Ooba, Yasunobu Ogura, Hisaya Tachibana, Hiroshi Ookawa, Toshihiro Fujii

**Affiliations:** 1 Department of Nephrology, Faculty of Medicine, University of Tsukuba, 1-1-1 Tennodai, Tsukuba, Ibaraki 305-8575 Japan; 2 Department of Nephrology, Mito Saiseikai General Hospital, Mito, Japan; 3 Department of Renal and Dialysis Medicine, Tsukuba Central Hospital, Ushiku, Japan; 4 Central Jin Clinic, Ryugasaki, Japan; 5 Present Address: Department of Nephrology, Ushiku Aiwa General Hospital, Ushiku, Japan; 6 Department of Nephrology, Tokyo Medical University Ibaraki Medical Center, Ami, Japan; 7 Nephrology Division, Department of Internal Medicine, JA Toride Medical Center, Toride, Japan; 8 Department of Nephrology, Ibaraki Prefectural Central Hospital, Kasama, Japan; 9 Moriya Keiyu Hospital, Moriya, Japan; 10 Department of Nephrology, Hitachi General Hospital, Hitachi, Japan; 11 Tsuchida Naika Hinyoukika Clinic, Hitachinaka, Japan; 12 Ohta Nephro Clinic, Hitachiohta, Japan

**Keywords:** Dialysis initiation, Prospective cohort study, Ibaraki prefecture

## Abstract

**Background:**

Patients with end-stage kidney disease (ESKD) face higher risks of life-threatening events including cardiovascular disease. Various risk factors are identified as agents influencing the life prognosis of ESKD patients. Herein, we evaluated the risk factors related to the outcomes of Japanese patients with dialysis induction. We present the study protocol, the patients’ baseline characteristics, and their outcomes.

**Methods:**

The Ibaraki Dialysis Initiation Cohort (iDIC) Study is a prospective multi-center cohort study in collaboration with 60 tertiary-care facilities in Ibaraki Prefecture, Japan. We collected baseline data from clinical records and analyzed blood and urine samples of these facilities’ patients with diabetic nephropathy, hypertensive nephrosclerosis, and chronic glomerulonephritis (CGN). The study’s primary outcome was the survival rate at 24 months after dialysis induction. We performed a Kaplan-Meier analysis for cumulative survival and a Cox proportional hazards analysis for all-cause mortality and hospitalization.

**Results:**

We analyzed 636 patients’ cases (424 males, 212 females, age 67.4 ± 13.1 yrs. [mean ± SD]). We compared the patients’ baseline data with those of similar cohort studies. As the primary kidney disease, 327 cases (51.4%) were diagnosed as diabetic nephropathy, 101 (15.9%) as hypertensive nephrosclerosis, and 114 (17.9%) as CGN. The mean serum creatinine value was 9.1 ± 2.9 mg/dL. The mean estimated glomerular filtration rate was 5.6 ± 1.8 mL/min/1.73m^2^. The cumulative survival rates at 6 months and 24 months after dialysis induction were 95.2 and 87.7%, respectively. The cumulative survival rate was significantly lower with increasing age. A Cox proportional hazards regression analysis demonstrated that high age was significantly associated with all-cause mortality.

**Conclusions:**

Regarding the clinical characteristics of these newly induced dialysis patients, the same trend as in other cohort studies was observed. Another study is underway to explore prognostic factors based on the iDIC Study’s findings.

## Background

Retarding the progression of chronic kidney disease (CKD) has been a primary endeavor of nephrologists for many years, and dialysis initiation has often been regarded as the main endpoint in this process. Patients with CKD stage G5D, often referred to as end-stage kidney disease (ESKD), have higher risks of cardiovascular disease (CVD) events [1]. Their prognosis is the worst of all CKD stages, sometimes progressing to death at an early stage of dialysis initiation. Various efforts have been made to prolong the healthy life expectancy of patients with ESKD, with numerous investigations into the primary causes of CKD, clinical manifestations, and clinical courses. This research has identified many risk factors for the speed of the progression of renal dysfunction, such as albuminuria. The accumulated experiences and observations have helped clarify the mechanisms underlying the deaths of some dialysis patients and have contributed to improvements in their healthy life expectancy.

Clinicians’ understanding of the progression of pre- and post-dialysis CKD has been growing. According to the results of the Dialysis Outcomes and Practice Patterns Study (DOPPS), Japanese hemodialysis patients had significantly better long-term survival than similar patients in Europe and the U.S., but their short-term (≤6-month) survival was not better [2]. Further research is thus necessary to explore the risk factors that are related to the outcomes of Japanese patients with dialysis initiation. In this context, we have planned a prospective multi-center cohort study in a local region, Ibaraki Prefecture, called the Ibaraki Dialysis Initiation Cohort (iDIC) Study, to analyze newly initiated dialysis patients’ baseline data obtained from clinical records and blood and urine samples. In this brief report, we present the study protocol, patient baseline characteristics, and outcomes of the iDIC Study.

## Patients and methods

### The basic research protocol of the iDIC study

The present study, i.e., the iDIC Study, was planned as a prospective multi-center cohort study in a specific region of Japan, i.e., Ibaraki Prefecture (one of Japan’s 47 prefectures). Sixty tertiary-care institutions (universities, referring hospitals, and dialysis clinics; see the Appendix) participated in this cohort study, enrolling consecutive patients with newly initiated dialysis and following each patient for 24 months. The inclusion criteria of this study were: (1) consecutive patients with newly initiated dialysis at any of the participating institutions from January 2013 to December 2015; and (2) patients who needed continuous maintenance dialysis at that institution. No exclusion criteria were set, and we did not conduct a sample-size calculation; this is because the registration in the study was of all of the cases of patients with newly initiated dialysis.

As the outcome assessment, the primary outcomes of this study were all-cause mortality, cardiovascular-related mortality, and infection-related mortality after dialysis initiation. The secondary outcomes were the rates of admission caused by infection, malignancy, cardiovascular-related events, and arteriovenous fistula-related events. This cohort study was performed in accord with the Declaration of Helsinki and the Ethical Guidelines for Epidemiological Research in Japan. Written informed consent was obtained from each participant for participation, publication, and availability of data and materials. The study protocol was approved by the ethics committee at each participating institution (the main institution being the University of Tsukuba Hospital, approval no. H24–116). The study was registered with the University Hospital Medical Information Network Clinical Trials Registry (UMIN000010806). Patient clinical records and blood and urine samples were collected at baseline. The patients’ entries, diagnoses of primary disease, and clinical records were provided by the responsible physician of each participating institution. The chief investigator of this cohort, the Consortium representative (KY), the officer of the research center (JU), and investigators at the top five institutions in terms of enrollment numbers (IE, TI, MK, YM, HK) comprised the study’s steering committee.

### The diagnosis of primary diseases

The primary cause of ESKD in each patient, e.g., diabetic nephropathy, chronic glomerulonephritis (CGN), or hypertensive nephrosclerosis, was diagnosed on the basis of the patient’s medical history and renal biopsy results. The diagnosis of primary disease including the pathological diagnosis of the renal biopsy was decided by the responsible physician of each participating institution. As the standard clinical practice, the clinical diagnosis of diabetic nephropathy was based on the following criteria: (1) a diabetes duration ≥10 years; (2) the confirmation of diabetic retinopathy (typically neo-vascular proliferative lesion) and neuropathy (typically sensory disorders of the distal portion of limbs); (3), the observation of overt proteinuria (typically nephrotic syndrome); and (4) the exclusion of other primary causes such as primary glomerulonephritis, and other secondary forms of glomerulonephritis including collagen disorder or vasculitis, hereditary kidney disease, cystic kidney disease, drug-induced kidney injury, and others.

The clinical diagnosis of hypertensive nephrosclerosis was based on the following criteria: (1) a hypertension duration ≥10 years; (2) the lack of overt proteinuria; (3) the confirmation of hypertensive retinopathy; and (4) the exclusion of other primary causes such as primary glomerulonephritis, other forms of secondary glomerulonephritis including diabetic nephropathy, collagen disorder or vasculitis, hereditary kidney disease, cystic kidney disease, drug-induced kidney injury, and others. In patients who had both diabetes and hypertension with no renal biopsy, the responsible physician of each participating institution diagnosed a single primary disease as in clinical practice.

### Baseline characteristics and comorbidities

Each patient’s clinical information and laboratory data were collected at study entry via a uniform questionnaire. The questionnaire was completed by the responsible physician of each participating institution. The baseline information included the patient’s age, gender, primary cause of ESKD, renal histological diagnosis, complications including diabetes (International Classification of Diseases, Tenth Revision [ICD-10] codes E10 to E14) and hypertension (ICD-10 codes I10 to I15), prior history of cardiovascular disease or malignancy, kidney disease in the family, regular medical checkup results, health insurance status, motive for consultation, the length of time between the referral and the initiation of dialysis, the estimated glomerular filtration rate (eGFR) value at three time points just before dialysis initiation, the length of time between surgery for an arteriovenous fistula to dialysis initiation, and the method of continuous dialysis treatment (hemodialysis, peritoneal dialysis or both). The patient’s physical status at the baseline included height, body weight, body mass index (BMI), blood pressure, and daily urine volume.

The laboratory data included (from blood) hemoglobin, hematocrit, urea nitrogen, creatinine, albumin, total cholesterol, triglyceride, HDL-cholesterol, LDL-cholesterol, hemoglobin A1c, glucose, uric acid, calcium, phosphate, beta 2-microgloblin, intact-parathyroid hormone, and whole-parathyroid hormone, and (from urine) protein, blood, urea nitrogen, and creatinine. The routine laboratory tests used for each entry were performed at each participating institution (without central collection), and thus the measurement methods used to obtain the above-listed laboratory values were not uniform among the 60 institutions.

Medication categories at dialysis initiation included angiotensin-converting enzyme inhibitors, angiotensin receptor blockers, beta-blockers, calcium channel blockers, statins, anti-platelet drugs including aspirin, active vitamin D analogues, erythropoiesis-stimulating agents, and insulin and other anti-diabetic agents. Prior treatments for primary glomerulonephritis included glucocorticoids, immunosuppressive reagents, and tonsillectomy. The qualitative data are presented as the number of cases and the rate (%). The quantitative data are presented as the mean value ± standard deviation (SD).

### Outcomes and statistical analysis

The primary endpoint of this study was the patients’ survival rate at 24 months after dialysis initiation. The secondary endpoint was the incidence of hospitalization events due to cardiovascular disease, infection, malignancy, and other causes. As is standard clinical practice, we defined ‘cardiovascular disease’ as coronary artery disease, cardiomyopathy, valvular heart disease, cerebrovascular disease, and peripheral vascular disease [3]. The infections included pulmonary infections (e.g., viral pneumonia, bacterial pneumonia, influenza, abscess of lung), genitourinary infections (e.g., pyelonephritis), gastrointestinal infections (e.g., appendicitis, diverticulitis, cholecystitis, cholangitis, peritonitis), peritonitis, soft-tissue infections (e.g., cellulitis), joint or bone infections (e.g., infective arthritis), and endocarditis [4]. The entries whose follow-up period was less than 3 months were excluded from outcome analysis. Almost entries who lost follow-up transferred to outpatient clinics that were out of this study group.

The patients’ cumulative survival, event-free cumulative survival, and cumulative incidence of hospitalization events were analyzed using the Kaplan-Meier method and compared using the log-rank test. We also analyzed cumulative survival as it related to the patients’ ages, classified in the following four groups: 0–39, 40–64, 65–84, and > 85 years old. We performed a Cox proportional hazards analysis using all-cause mortality and all-cause hospitalization events as the dependent variable. We selected the independent variables that have been reported to be associated with the prognosis of patients with newly initiated dialysis [5]. The independent variables were age, BMI, hemoglobin, eGFR, serum albumin, serum corrected calcium, serum phosphate and intact-parathyroid hormone. There was no multicollinearity among the independent variables. Probability (*p*)-values < 0.05 were considered significant. The IBM SPSS software package ver. 22 was used for all statistical analyses.

## Results

A total of 636 patients (424 males, 212 females) registered from January 2013 to December 2015 were enrolled in this study (Table 1). Their ages ranged from 17 to 93 years old (mean ± SD, 67.4 ± 13.1 years). At their dialysis initiation, 12.8% of the patients had a history of renal biopsy, 40.3% had a medical checkup history, 58.5% presented with diabetes, and 93.8% presented with hypertension. Complications prior to dialysis initiation included myocardial infarction (9.6%), stroke (15.5%), peripheral artery disease (9.9%), and malignancy (15.0%). Regarding the primary kidney diseases, 327 cases (51.4%) were diagnosed as diabetic nephropathy, 101 cases (15.9%) as hypertensive nephrosclerosis, and 114 cases (17.9%) as CGN. Serum creatinine was 9.1 ± 2.9 mg/dL, with the eGFR of 5.6 ± 1.8 mL/min/1.73m^2^ at the dialysis initiation.

**Table 1:** Comparion of clinical characteristics at entry in dyalisis indcution cohort studies

	iDIC study	AICOPP	JSDT	USRDS
year	2013–2015	2011–2013	2006	2013–2015
Number	636	1524	9689	363,307
Dialysis modality (HD/PD/transplant)	614/22/0	1409/105/0	8702/443/0	319,309/33,861/9325
Age (years old)	67.4 ± 13.1	67.5 ± 13	66.6 ± 13.2	62.5
Gender (male/female)	424/212	1032/492	6241/3448	210,785/152,522
Renal biopsy (%)	12.8	NA	N/A	NA
Medical checkup history (%)	40.3	NA	NA	NA
Diabetes at dialysis initiation (%)	58.5	53.3	58.8	NA
Hypertension at dialysis initiation (%)	93.8	NA	NA	NA
Prior myocardial infarction (%)	9.6	15.8	8.0	NA
Prior stroke (%)	15.5	13.7	15.0	NA
Prior peripheral artery disease (%)	9.9	4.7	NA	NA
Prior malignancy (%)	15.0	10.6	7.3	NA
Family history of kidney disease (%)	10.3	NA	NA	NA
Primary kidney disease (%)				
Diabetic nephropathy	51.4	43.2	42.9	44.8
Hypertensive nephrosclerosis	15.9	25.3	9.4	28.5
Chronic glomerulonephritis	17.9	14.7	25.6	7.5
Autosomal dominant polycystic kidney disease	2.5	3.3	2.4	2.2
Rapidly progressive glomerulonephritis	2.5	NA	1.2	NA
Other disease	6.0	13.5	8.6	17.0
Unknown cause	3.8		9.9	2.4
Body mass index (kg/m^2^)	24.3 ± 4.5	23.5 ± 4.4	NA	29.6
Systolic blood pressure (mmHg)	150.6 ± 26.5	151.1 ± 26.0	153 ± 28	NA
Diastolic blood pressure (mmHg)	77.8 ± 16.3	77.1 ± 15.0	78 ± 16	NA
Hemoglobin (g/dL)	9.0 ± 1.8	9.4 ± 1.5	8.4 ± 1.6	9.4
Serum creatinine (mg/dL)	9.1 ± 2.9	9.0 ± 3.2	8.2 ± 3.3	6.4
Estimated glomerular filtration rate (mL/min/1.73m^2^)	5.6 ± 1.8	5.6 ± 2.2	5.5 ± 3.3	9.8
Serum uric acid (mg/dL)	8.5 ± 8.6	8.8 ± 2.4	NA	NA
Serum albumin (g/dL)	3.5 ± 2.6	3.2 ± 0.6	3.3 ± 0.6	3.2
Serum total cholesterol (mg/dL)	158.5 ± 48.0	162 ± 44.3	NA	155.5
Serum corrected calcium (mg/dL)	8.5 ± 1.0	8.6 ± 1.1	8.6 ± 1.1	NA
Serum phosphate (mg/dL)	6.2 ± 1.9	6.4 ± 1.9	5.8 ± 1.9	NA
Hemoglobin A1c (%)	5.9 ± 0.9	5.8 ± 0.9	NA	6.7
Serum β2-microglobulin (mg/L)	26.8 ± 52.5	NA	NA	NA
Intact-parathyroid hormone (pg/ml)	289.5 ± 224.5	353 ± 292.4	NA	NA
Cumulative survival rate after 6 months (%)	95.2%	NA	NA	NA
Cumulative survival rate after 12 months (%)	93.7%	NA	87.4%	80.8%
Cumulative survival rate after 24 months (%)	87.7%	NA	NA	70.0%

*Abbreviations*: *iDIC Study* the Ibaraki Dialysis Initiation Cohort Study, *AICOPP* Aichi Cohort Study of Prognosis in Patients Newly Initiated Into Dialysis, *JSDT* The Japanese Society for Dialysis Therapy, *USRDS* the United States Renal Data System, *HD* Hemodialysis, *PD* Peritoneal dialysis, *NA* not available

To analyze primary and secondary endpoints, 542 entries following for 3 months and more were investigated. The cumulative survival of the total patient series was determined. Table 2 provides the cumulative survival rate, the event-free survival rate, and the incidence of hospitalization events at 6, 12 and 24 months after the patients’ dialysis initiation. The cumulative survival rates at 6, 12 and 24 months after dialysis induction were 95.2, 93.7 and 87.7%, respectively. The event-free cumulative survival rates at 6, 12 and 24 months after dialysis induction were 80.1, 69.3 and 50.2%, respectively. The incidence of hospitalization events at 6 months was 16.8%, and that at 12 months was 43.4%.

**Table 2:** Cumulative survival rate, event free survival rate and incidence of hospitalization event after 6, 12 and 24 months of dialysis induction

	6 months (%)	12 months (%)	24 months (%)
Cumulative survival rate	95.2%	93.7%	87.7%
Event free cumulative survival rate	80.1%	69.3%	50.2%
Cumulative incidence of hospitalization event	16.8%	27.2%	43.4%
Cumulative incidence of hospitalization related with cardiovascular event	2.4%	4.4%	13.2%
Cumulative incidence of hospitalization related with malignancy	1.8%	3.0%	4.6%
Cumulative incidence of hospitalization related with infectious event	2.4%	4.4%	7.9%

**Fig. 1:**
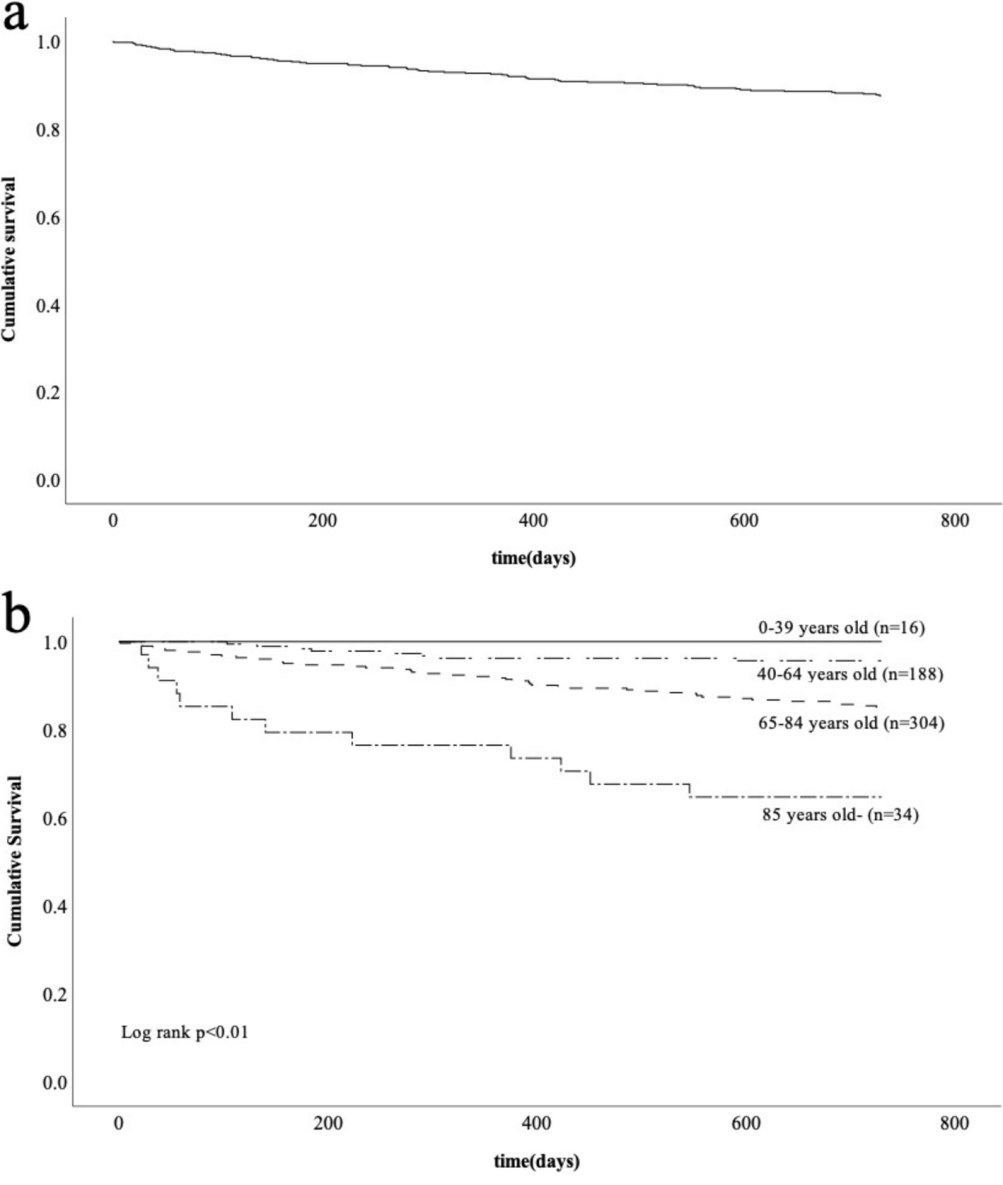
Kaplan-Meier curves for the cumulative survival of the patients with dialysis initiation (*n* = 542). **a** Kaplan-Meier curves for the cumulative overall survival. **b** The cumulative survival of the patients in each age group. The cumulative survival rate was significantly lower with higher age (*p* < 0.01)

Figure 1a provides the Kaplan-Meier curves for the cumulative overall survival of the total patient population. The results of the comparison of the clinical information of the four age groups are listed in Table 3: 0–39 years old (*n* = 16), 40–64 years old (*n* = 188), 64–85 years old (*n* = 304), and > 85 years old (*n* = 34). The two older patient groups had significantly lower rates of renal biopsy (*p* < 0.01) and significantly lower rates of a medical checkup history (*p* < 0.01). In the 0- to 39-year-old group, the complication rate of diabetes mellitus was the same as the rate of diabetic nephropathy as the primary disease.

**Table 3:** The comparison of clinical records among age groups

	0–39 years old	40–64 years old	65–84 years old	over 85 years old	*P*
Number	16	188	304	34	
Age (years old)	31.9 ± 7.3	55.4 ± 6.9	73.4 ± 5.9	87.3 ± 2.2	
Gender (male/female)	12/4	133/55	203/101	16/18	0.51
Renal biopsy (%)	37.5	18.8	9.3	0.0	< 0.01
Medical checkup history (%)	66.7	57.0	29.8	12.5	< 0.01
Diabetes at dialysis initiation (%)	25.0	65.6	59.8	38.2	< 0.01
Hypertension at dialysis initiation (%)	93.8	97.3	93.7	88.2	0.12
Prior myocardial infarction (%)	0.0	8.1	12.1	8.8	0.28
Prior stroke (%)	0.0	12.1	18.4	8.8	0.06
Prior peripheral artery disease (%)	0.0	7.4	9.3	12.9	0.45
Prior malignancy (%)	0.0	7.3	18.7	11.8	< 0.01
Family history of kidney disease (%)	13.3	12.9	10.2	7.7	0.80
Primary kidney disease (%)					
Diabetic nephropathy	25.0	61.2	49.7	32.4	< 0.01
Hypertensive nephrosclerosis	0.0	8.5	17.8	38.2	
Chronic glomerulonephritis	43.8	18.6	16.4	11.8	
Autosomal dominant polycystic kidney disease	0.0	5.3	1.3	0.0	
Rapidly progressive glomerulonephritis	0.0	1.6	3.0	5.9	
Other disease	25.0	2.7	6.9	8.8	
Unknown cause	6.3	2.1	4.9	2.9	
Body mass index (kg/m^2^)	27.0 ± 9.4	25.9 ± 5.0	23.7 ± 3.7	22.3 ± 3.3	< 0.01
Systolic blood pressure (mmHg)	157.8 ± 29.9	154.1 ± 24.1	149.2 ± 26.5	141.4 ± 32.4	0.01
Diastolic blood pressure (mmHg)	99.1 ± 16.5	82.5 ± 16.3	75.0 ± 15.2	69.2 ± 14.1	< 0.01
Hemoglobin (g/dL)	10.0 ± 4.5	9.0 ± 1.6	8.9 ± 1.8	9.2 ± 1.7	0.53
Serum creatinine (mg/dL)	11.9 ± 3.7	10.2 ± 3.1	8.6 ± 2.6	7.2 ± 2.3	< 0.01
Estimated glomerular filtration rate (mL/min/1.73m^2^)	4.2 ± 1.3	4.9 ± 1.6	5.8 ± 1.8	6.7 ± 2.0	< 0.01
Serum uric acid (mg/dL)	9.4 ± 2.2	8.2 ± 2.2	7.8 ± 2.3	7.8 ± 2.4	0.03
Serum albumin (g/dL)	3.5 ± 0.7	3.3 ± 0.6	3.3 ± 0.6	3.1 ± 0.7	0.18
Serum total cholesterol (mg/dL)	192.2 ± 68.7	163.2 ± 52.8	154.3 ± 44.5	163.4 ± 47.1	0.08
Serum corrected calcium (mg/dL)	8.2 ± 1.1	8.3 ± 1.0	8.6 ± 1.1	8.8 ± 0.9	< 0.01
Serum phosphate (mg/dL)	6.8 ± 2.2	6.7 ± 2.0	6.1 ± 1.8	5.4 ± 1.4	< 0.01
Hemoglobin A1c (%)	5.4 ± 1.4	5.9 ± 0.9	5.9 ± 0.9	5.7 ± 0.7	0.04
Serum β2-microglobulin (mg/L)	17.8 ± 4.9	25.1 ± 43.2	29.0 ± 61.4	19.6 ± 7.1	0.07
Intact-parathyroid hormone (pg/ml)	494.3 ± 309.9	365.2 ± 286.4	241.7 ± 172.9	222.0 ± 58.9	< 0.01

Among the 40- to 84-year-old patients, > 50% had diabetes mellitus, which is higher than the rate of diabetic nephropathy as the primary disease in these patients. Malignancies were less common in the patients ≤39 years old. The complication rate of malignancy increased with age in the patients 40–84 years old and was rather lower in the patients > 85 years old compared to the patients aged 65–84 (*p* < 0.01). With the patients’ advancing age, the rate of nephrosclerosis increased but the rate of CGN decreased. The rate of diabetic nephropathy increased up to the age of 64 years but decreases slightly with age in the patients > 65 years old (*p* < 0.01).

The patients’ BMI decreased significantly with age (*p* < 0.01), as did their systolic blood pressure (*p* = 0.01); the eGFR values increased significantly with age (*p* < 0.01). The group of 0- to 39-year-old patients had the highest values of serum uric acid, followed by the 40–64 year-old group, and then the > 65-year-olds (*p* = 0.03). The serum corrected calcium values increased significantly with age (*p* < 0.01). The serum phosphate values decreased significantly with age (*p* < 0.01), as did the intact-parathyroid hormone values (*p* < 0.01).

As shown by the Kaplan-Meier curves for the cumulative survival stratified by age group (Fig. 1), the cumulative survival rate was significantly lower with increasing age (*p* < 0.01). The results of the Cox proportional hazards regression analysis for all-cause mortality are provided in Table 4a: high age was significantly associated with all-cause mortality (hazard ratio: 1.07, 95% confidence interval: 1.01–1.13, *p* = 0.02). The Cox proportional hazards regression analysis for the development of all-cause hospitalization events revealed that none of the variables examined was associated with all-cause hospitalization (Table 4b).

**Table 4:** COX proportional hazards analysis with all-cause mortality and hospitalization as the dependent variable

Independent variable	Hazard ratio	95.0% confidence interval of hazard ratio		*P*
		Lower limit	Upper limit	
a. All-cause mortality				
Age	1.07	1.01	1.13	0.02
Body mass index	0.88	0.77	1.00	0.06
Hemoglobin	0.87	0.64	1.18	0.36
Estimated glomerular filtration rate	1.09	0.82	1.45	0.57
Serum albumin	0.76	0.32	1.79	0.52
Serum corrected calcium	1.09	0.69	1.73	0.70
Serum phosphate	1.17	0.87	1.59	0.30
Intact-parathyroid hormone	1.00	1.00	1.00	0.20
b. All-cause hospitalization				
Age	1.02	0.99	1.04	0.18
Body mass index	0.99	0.94	1.04	0.68
Hemoglobin	0.98	0.86	1.11	0.74
Estimated glomerular filtration rate	0.95	0.81	1.12	0.55
Serum albumin	0.71	0.47	1.08	0.11
Serum corrected calcium	1.00	0.78	1.29	0.99
Serum phosphate	0.90	0.77	1.06	0.20
Intact-parathyroid hormone	1.00	1.00	1.00	0.23

## Discussion

We have established a prospective multi-center cohort study in a local region of Japan, Ibaraki Prefecture, to analyze the clinical information of newly initiated dialysis patients. As the focal point of this research, blood and urine samples were collected for the investigation of potential biological markers of life-threatening event risks. We have described the protocol of this (iDIC) study and the patients’ baseline clinical data and major outcomes.

The baseline data of our enrolled patients show a tendency in the following findings that is similar to that of three previous studies that analyzed the clinical characteristics of new-initiation dialysis patients: the Aichi Cohort Study of Prognosis in Patients Newly Initiated into Dialysis (AICOPP) [6], a report by the Japanese Society for Dialysis Therapy (JSDT) [7], and the U.S. Renal Data System (USRDS) [8] (Table 1). Those studies had more men than women with an average age in the 60s, showing a high prevalence of diabetic nephropathy as the primary kidney disease, followed by CGN or hypertensive nephrosclerosis. Concerning prior complications, the percentage of myocardial infarction in the present study is lower than those of the other studies, whereas stroke and malignancy accounted for a larger percentage compared to the other studies. Our patients’ laboratory data including hemoglobin, serum uric acid, serum albumin, serum total cholesterol, serum corrected calcium, and serum phosphate (other than serum creatinine and eGFR) were essentially at the same levels as those of the previous studies.

Most strikingly, the serum creatinine level and eGFR of the present patients were close to those of the previous two studies conducted in Japan and lower than that of the USRDS patients, supporting the hypothesis that the timing of dialysis initiation has been later in Japan compared to the U.S. However, a notable point is that even in the U.S., the mean eGFR at the initiation of dialysis has been decreasing in recent years: 9.7 mL/min/1.73m^2^ was recorded in 2016, down from a peak of 10.4 mL/min/1.73m^2^ in 2010 [9], reflecting the growing consensus that the initiation of dialysis at higher eGFR values has not shown a survival benefit.

Our present analyses revealed the survival rate of 636 patients with dialysis initiation. Nationwide survey data in 2015 aggregated by the JSDT demonstrated that the 2-year survival rate of Japanese patients with dialysis initiation was 83.5%. The present study’s 2-year survival rate of patients with dialysis initiation, i.e., 87.7%, is comparable to that of the JSDT data [7]. By contrast, the U.S. nationwide survey data in 2015 reported by the USRDS showed a corresponding 2-year survival rate at 69.4%; the 2-year survival rate of our present iDIC study patients is clearly higher than that of the USRDS data [8]. The DOPPS report showed that U.S. dialysis patients had a higher mortality rate than Japanese dialysis patients because the U.S. dialysis patients were older than the Japanese patients (60.5 ± 15.5 years vs. 58.6 ± 12.5 years old) and also had higher prevalences of diabetes, coronary artery disease, congestive heart failure, peripheral vascular disease, and cerebrovascular disease [9]. Thus, the prognosis of new-initiation dialysis patients differs among various countries.

We noted above that the patients’ life expectancy was associated with their age at the initiation of dialysis. In the present population, the cumulative survival rate of the patients with dialysis initiation depended on their age. The patients’ age was the only risk factor that was closely associated with survival in both the univariate and multivariate statistical analyses. The JSDT analysis also demonstrated that every additional year of age increased the risk of death among patients with dialysis initiation by 1.055 (95% confidence interval: 1.052–1.058) [5]. The USRDS study indicated that the 2-year survival rate of patients with dialysis initiation tended to decrease with increasing age [8]. The age dependency of the life expectancy of new-initiation dialysis patients has thus shown a common trend globally.

The limitations of our study include potential variations in measurement protocols and diagnosis standards, as different expert physicians in each facility were responsible for the primary disease diagnosis of CGN, diabetic nephropathy, and hypertensive nephrosclerosis and may have interpreted their diagnoses differently. The data were limited to patients in a specific region of Japan, and they may not be generalizable to other populations. The observation period of our study was limited to 2-year observations, and the related research made it difficult to investigate longer-term prognoses. A follow-up of the patients’ samples after the initiation of dialysis was not performed, and we were thus unable to analyze changes in candidate biomarkers’ values as risk factors.

## Conclusions

We have presented the study protocol, baseline patient characteristics, and major outcome of the iDIC Study. The trends in the clinical characteristics of this study’s new-initiation dialysis patients were the same as those reported in the other cohort studies. An additional study is underway to explore prognostic factors based on the iDIC Study’s findings.

## Data Availability

The datasets used and/or analyzed in this study are available from the corresponding author on reasonable request.

## Abbreviations

AICOPP: Aichi Cohort Study of Prognosis in Patients Newly Initiated Into Dialysis
CGN: Chronic glomerulonephritis
CKD: Chronic kidney disease
CVD: Cardiovascular disease
DOPPS: Dialysis Outcomes and Practice Patterns Study
ESKD: End-stage kidney disease
eGFR: Estimated glomerular filtration rate
ICD-10: The International Classification of Diseases, Tenth Revision
iDIC: Ibaraki Dialysis Initiation Cohort
JSDT: The Japanese Society for Dialysis Therapy
USRDS: The United States Renal Data System
